# Homer1 ameliorates ischemic stroke by inhibiting necroptosis-induced neuronal damage and neuroinflammation

**DOI:** 10.1007/s00011-023-01824-x

**Published:** 2023-12-13

**Authors:** Weihao Lv, Qianqian Zhang, Yuanming Li, Dan Liu, Xiuquan Wu, Xin He, Yuanyuan Han, Xiaowei Fei, Lei Zhang, Zhou Fei

**Affiliations:** 1 Department of Neurosurgery, Xijing Hospital, Air Force Medical University, No. 127, Changle West Road, Xincheng District, Xi’an, Shaanxi 710032 China; 2 Department of Respiratory Medicine, Lanzhou University Second Hospital, Lanzhou, 730070 China; 3 Department of Neurology, Gansu Province Central Hospital, Lanzhou, 730070 China

**Keywords:** Homer1, Ischemic stroke, Necroptosis, Neuroinflammation

## Abstract

**Objective:**

Proinflammatory necroptosis is the main pathological mechanism of ischemic stroke. Homer scaffolding protein 1 (Homer1) is a postsynaptic scaffolding protein that exerts anti-inflammatory effects in most central nervous system diseases. However, the relationship between Homer1 and proinflammatory necroptosis in ischemic stroke remains unclear.

**Aim:**

This study aimed to investigate the role of Homer1 in ischemia-induced necroptosis.

**Methods:**

C57BL/6 mice were used to establish a model of permanent middle cerebral artery occlusion model (pMCAO). Homer1 knockdown mice were generated using adeno-associated virus (AAV) infection to explore the role of Homer1 and its impact on necroptosis in pMCAO. Finally, Homer1 protein was stereotaxically injected into the ischemic cortex of Homer1^flox/flox^/Nestin-Cre ^+/−^ mice, and the efficacy of Homer1 was investigated using behavioral assays and molecular biological assays to explore potential mechanisms.

**Results:**

Homer1 expression peaked at 8 h in the ischemic penumbral cortex after pMCAO and colocalized with neurons. Homer1 knockdown promoted neuronal death by enhancing necroptotic signaling pathways and aggravating ischemic brain damage in mice. Furthermore, the knockdown of Homer1 enhanced the expression of proinflammatory cytokines. Moreover, injection of Homer1 protein reduced necroptosis-induced brain injury inhibited the expression of proinflammatory factors, and ameliorated the outcomes in the Homer1^flox/flox^/Nestin-Cre^+/−^ mice after pMCAO.

**Conclusions:**

Homer1 ameliorates ischemic stroke by inhibiting necroptosis-induced neuronal damage and neuroinflammation. These data suggested that Homer1 is a novel regulator of neuronal death and neuroinflammation.

**Supplementary Information:**

The online version contains supplementary material available at 10.1007/s00011-023-01824-x.

## Introduction

Acute ischemic stroke accounts for 80–85% of all strokes [1], with high mortality and disability rates [2–4], making it a major cause of human health. Currently, treatment measures for acute ischemic stroke primarily include surgery and medication [5], but some patients still have poor prognoses [6]. The main mechanisms underlying ischemic damage after acute ischemic stroke include neuronal necrosis [7], inflammatory response [8], and oxidative stress [9]. Therefore, reducing neuronal death in the ischemic penumbra and improving the inflammatory response are the main directions of preclinical research.

Tumor necrosis factor-α (TNF-α) can trigger a "programmed" form of necrosis (i.e., necroptosis) by binding to the receptor TNF-RI [10]. Necroptosis is a proinflammatory form of cell necrosis with regulatable signal transduction [11, 12], and has similarities and differences with necrosis and apoptosis. Mechanistically, necroptosis is triggered by a signaling cascade consisting of receptor-interacting protein kinase 1 (RIPK1), receptor-interacting protein kinase (RIPK3), and mixed lineage kinase domain-like protein (MLKL) [13]. MLKL is the main executor and hallmark molecule in the development of necroptosis [14]. This proinflammatory form of cell death plays an important role in the pathogenesis of ischemic stroke [15, 16].

Homer scaffolding protein1 (Homer1), a postsynaptic scaffold protein, is primarily expressed in the nervous system, although it is slightly expressed in the myocardial and skeletal muscles [17]. Preclinical studies have shown that Homer1 can inhibit changes in calcium ions and inflammation in the dorsal root ganglion, and ameliorate neuropathic pain [18]. Homer1 can also inhibit neuroinflammation in the bleeding site after cerebral hemorrhage, reduce cell death, and ameliorate the functional recovery of Homer1^flox/flox^/Nestin-Cre^+/−^ mice [19]. However, the relationship between Homer1 and proinflammatory necroptosis in acute ischemic stroke remains unclear.

In this study, we found that, in the ischemic penumbra cortex, the expression of Homer1 initially increased and then decreased during the early stages of ischemic brain injury. Knockdown of Homer1 aggravates ischemic brain injury. Furthermore, in the conditioned knockout of *Homer1* mouse model of pMCAO, Homer1 protein ameliorated brain damage by inhibiting neuronal death induced by necroptosis and the production of proinflammatory factors.

## Material and methods

### Experimental animal

All animal experiments were performed in accordance with the protocols approved by the Institutional Ethics Committee of Xijing Hospital. All experimental procedures were approved by the Institutional Animal Care and Use Committee of Air Force Medical University (approval no. IACUC-20220630). All animal experiments were designed according to the 3R principle. The 6–8-week C57BL/6 J male mice and Homer1^flox/flox^/Nestin-Cre^+/−^ mice (25 ± 2 g each) were purchased from the Shanghai Model Organisms Center, Inc. (Shanghai, China). There were no significant differences in age or weight among the different groups of mice.

## Experimental design

First, Homer1 expression was assessed in the sham group and at different time points in the pMCAO group. The mice were randomly divided into four groups: sham, 4 h, 8 h, and 12 h after pMCAO. The ischemic penumbral cortex from each group was collected for western blot (WB) analysis (*n* = 6/each group). Cellular localization of Homer1 was detected using double immunofluorescence staining (sham and 8 h MCAO groups, *n* = 6/each group).

In the second step, to study the effects of Homer1, mice were randomly divided into sham, pMCAO, and Homer1-KD groups. Each group (*n* = 6) was euthanized 8 h after pMCAO. The brain tissue of each group was used for WB, expression of neuroinflammation associated with necroptosis, and ELISA (*n* = 6/each group). The brain tissues of each group were used for immunohistochemistry (*n* = 6/each group). Each group of mice was scored for behavior before anesthesia (*n* = 10/each group). The brain tissues of each group were used to detect brain water content and for TTC staining (*n* = 6/each group).

In the third step, to explore the effects of treatment with Homer1 Protein, Homer1^flox/flox^/Nestin-Cre^+/−^ mice were randomly divided into two groups: one group was injected with Homer1 protein after pMCAO (defined as the Homer1 Protein group); The other group was injected with PBS after pMCAO (defined as the PBS group). At 8 h after pMCAO, all the mice were anesthetized and sacrificed. Brain tissues of six mice in each group were used for WB, mouse cytokine array detection, ELISA, immunohistochemistry, and TTC staining (*n* = 6/each group). Each group of mice was scored for behavior before anesthesia (*n* = 10/each group). Furthermore, 15 Homer1^flox/flox^/Nestin-Cre^+/−^ mice in each group were used to monitor the length of survival.

### Plasmid, adeno-associated virus, and Homer1 protein

Primary cortical neurons were transfected with plasmids by using jetPRIME Buffer (#201000003; Polyplus) reagent to stably knock down Homer1 (Homer1-KD) (pCDNA3.1-U6-shRNAHomer1-ZsGreen). The plasmid transfection process was strictly according to the protocol manual.

Mice were infected with adeno-associated virus (AAV) to stably knock down Homer1 (AAV2/9-U6-shRNAHomer1WPRE) in the right cortex. AAVs and plasmids were constructed with assistance from Hanbio Co., Inc. (Shanghai, China). The siRNA-target sequence was 5′-GCATTGCCATTTCCACATA-3′.

As described previously [19], stereotactic injection technology was been applied to the injection of AAV and Homer1 proteins. Firstly, the scalp of the experimental animal was disinfected and cleaned, and the surface of the animal's skull was exposed. According to the brain stereotactic map, the injection position (1.43 mm posterior and 3 mm lateral to the bregma) was determined using the bregma as the coordinate origin. The syringe was inserted into the brain (depth, 2.0 mm from the bone surface). Next, 2 μL of AAV wasere injected within 10 min. The syringe was withdrawn after 10 min. After surgery, the scalp was sutured and disinfected.

In the Homer1^flox/flox^/Nestin-Cre^+/−^ conditional knockout mice, PBS (20 μL) and Homer1 protein (ORIGENE, TP760819) (1 μg, dissolved in 20 μL PBS) were injected in situ (1.43 mm posterior and 3 mm lateral to the bregma) (depth, 2.0 mm from the bone surface) 5 min after pMCAO. Then, 20 μL of PBS/Homer1 protein was injected within 10 min. The syringe was withdrawn after 10 min. After surgery, the scalp was sutured and disinfected.

### MCAO model

Focal cerebral ischemic injury was induced by pMCAO in mice using the intraluminal filament technique as described previously [20]. Briefly, after the animal was initially anesthetized by inhaling 5% isoflurane, surgical procedures were performed and sedation was maintained by inhaling 2% isoflurane. The superficial fascia was cut to expose the right sternocleidomastoid muscle. Blunt separation was performed between the sternocleidomastoid and anterior cervical muscle groups to expose the carotid sheath. The common carotid artery (CCA) and vagus nerve were dissociated with a minute glass needle until CCA bifurcation. The internal carotid artery (ICA) was located posterolaterally in the supine position, and the external carotid artery (ECA) close to the medial trachea was observed. Three lines were attached to the CCA; the lowest ligature was tied to block the forward blood flow of the right CCA, and the uppermost ligature was gently tied to avoid blood reflux and release of the middle suture. A small incision was made with ophthalmic scissors 5 mm above the bottom ligation line, a nylon suture coated with silicone was inserted, and the second ligation line was gently tied. The top ligation line was released, and the nylon suture line was adjusted to enter the ICA. In the sham group, the insertion depth was less than 10 mm, and the other treatments remained unchanged. Throughout the entire surgical procedure, a pulse oximeter (SurgiVet, model V3304; Waukesha, WI, USA) was used to monitor blood oxygen saturation and a heating blanket was used to maintain body temperature between 36.5 °C and 37.5 °C.

### Primary neuron culture and oxygen–glucose deprivation (OGD) treatment

Cortical neurons were prepared from the cortex of embryonic day 17 (E17) C57BL/6 mice as previously described [21]. The cerebral cortex of fetal mice was taken out under sterile conditions and digested with 0.25% Trypsin–EDTA for 10 min. After filtration, cells were resuspended in Dulbecco’s modified Eagle medium (DMEM)/F-12 medium with 15% fetal bovine serum (FBS) and 1% penicillin/streptomycin, then inoculated into 24-well plates coated with polylysine, and incubated in a 37 ℃, 5% CO_2_ incubator for 4 h. After cell adhesion, the culture medium was replaced with neurobasal medium (21103049; Thermo Fisher Scientific) supplemented with B-27(12587010; Thermo Fisher Scientific) and glutamine (35050061; Thermo Fisher Scientific). The cells were then incubated in a CO_2_ incubator for 48 h, washed with PBS, and the medium was changed. After 24 h, half of the medium was changed and cytarabine was added to inhibit the growth of glial cells. After 48 h, the medium was replaced completely. Afterward, the medium was changed every 3 days, and the experiment was conducted approximately 8 days later. Primary neurons were identified using morphological analysis and NeuN staining. We strictly followed the extraction process of primary neurons and the purification method. In the stable stage of primary neuronal cells, we detected the percentage of NeuN-positive cells using immunofluorescence and found that the purity of neurons was above 98%, and GFAP-positive glial cells were almost undetectable after being inhibited by cytarabine.

To mimic ischemic injury in vitro, the medium was replaced with glucose-free neurobasal medium, and primary neuronal cells were then cultured at 37 °C in an OGD chamber containing 5% CO_2_ and 95% N_2_.

### TTC staining and brain tissue water content detection

Brain specimens were collected at specific time points. Coronary sections were performed at an interval of 2 mm through brain matrices. The brain sections were placed in a 10 cm culture dish and incubated with phosphate-buffered saline containing 2% TTC at 37 ℃ for 30 min. ImageJ analysis software was used to analyze the scanned images, and the percentage of infarcts was calculated as the percentage of contralateral structures.

Water content detection in brain tissue: The brain tissue of six mice in each group was removed from the olfactory bulb, cerebellum, and lower brainstem and weighed as the wet mass of the brain. After baking for 72 h, the sample was weighed to obtain the dry mass. Brain water content (%) = [(wet mass-dry mass)/wet mass] × 100%.

### Behavioral testing

The animal experiments involved two persons: one who was in charge of animal handling, and the other who performed the behavioral tests.

#### Longa score

As described previously [19], the scoring criteria were as follows: no neurological deficits, 0 points; inability to fully extend the paralyzed front paw, 1 point; rotation towards the paralyzed side while walking, 2 points; tilting towards the paralyzed side while walking, 3 points; and inability to walk automatically and experiencing loss of consciousness, 4 points.

#### Corner test

As described previously [22], this experimental method detects the comprehensive sensory and motor functions of mice, including the stimulation of tentacles (sensation/defect) and hind leg standing up (motor response). When the mouse entered the depth of the angle, its tentacles on both sides first touched the wooden board. The mouse stood up with its hind legs facing forward and then turned around to face outside the angle. Only by fully standing up with the hind legs at the included angle and then turning around was it possible to complete the corner experiment. Each mouse was tested ten times, and the score was calculated as the number of turns to the healthy side/total number of turns × 100%.

#### Rota-rod test

As described previously [23], the rotating rod was placed horizontally and accelerated from 4 to 40 rpm for 5 min. Before the operation, the mice were trained for 3 days at different rpm separately with an interval of 15 min. This experiment requires animals to have complete motor skills and sensory–motor integration, and the time the animals stay on the rotating stick can be used as an indicator of the motor function of the experimental animals.

### Mouse genotype detection

As described previously [19], the tail of the mouse was cut and digested to extract DNA. Afterward, the PCR reaction was carried out according to the protocol of the One-Step Mouse Genotyping Kit (Vazyme). Identification of Homer1-LoxP-1(wild type: 261 bp; mutant: 324 bp): Primer 1 (forward): 5′-AGCTGCTTGTATAGTCTGAGTGC-3′, Primer 2 (reverse): 5′-GGCTTTCCTCAATAATGAACTG-3′; Homer1-LoxP-2 (wild type: 129 bp; mutant: 200 bp): Primer 1 (forward): 5′-GTTGCAGCACTGTATGACTTGATAA-3′, Primer 2 (reverse): 5′-CTTCAATTCTACTGCATGGACTGT-3′; identification of Nestin-Cre (wild type: 246 bp; mutant: 150 bp): Primer 1 (wild type): 5′-TTGCTAAAGCGCTACATAGGA-3′, Primer 2 (mutant): 5′-CCTTCCTGAAGCAGTAGAGCA-3′, Primer 3 (common): 5′-GCCTTATTGTGGAAGGACTG-3′.

### Mouse cytokine array

The levels of inflammatory factors and cytokines were measured in the ischemic penumbra cortex. A mouse cytokine array was provided by RayBiotech (QAM-CAA-1000, USA). Experimental procedures were performed in strict accordance with the manufacturer’s instructions.

### Nissl staining

As described previously [19], frozen sections were washed three times with PBS for 5 min each time and stained with 1% toluidine blue (Beyotime, Shanghai) at 56 °C for 20 min. Afterward, the frozen sections were soaked in 75% alcohol for 1 min and differentiated in 95% alcohol. Slices were dehydrated in anhydrous ethanol and soaked in xylene for 5 min. Finally, the frozen sections were sealed with neutral resin.

### TUNEL staining

The experimental procedure for TUNEL staining was strictly carried out in accordance with the manufacturer's instructions (Beyotime, Shanghai, China). Dead cells were observed and stained using a fluorescence microscope (Olympus IX71; Olympus Corporation, Tokyo, Japan).

### Immunofluorescence assay

As described previously [19], frozen sections were first permeabilized with 0.3% Triton-X-100 for 30 min and blocked with 10% goat serum in PBS for 2 h. Primary antibodies were incubated overnight at 4 °C and washed several times with PBS, sections were stained with secondary antibodies for 1 h, and nuclei were stained with DAPI (S2110, Solarbio). The following antibodies were used: mouse anti-NeuN (1:100; ab104224; Abcam; UK), mouse anti-GFAP (1:100; ab4648; Abcam; UK), mouse anti-Iba1(1:100; ab283319; Abcam; UK); rabbit anti-Homer1 (1:200; #ab184955; Abcam; UK), rabbit Phospho-MLKL (1:200; #AF3846; Affinity; USA); Goat Anti-Mouse IgG H&L, Alexa Fluor 488 (1:200; Abcam); goat anti-rabbit IgG (H + L) highly cross-adsorbed secondary antibody, Alexa Fluor Plus 488 (1:1000; A-11034; Invitrogen; USA), and goat anti-mouse IgG (H + L) highly cross-adsorbed secondary antibody, Alexa Fluor Plus 555 (1:1000; A32727; Invitrogen; USA).

### Western blot analysis

As described previously [24], The ischemic penumbra cortex was thoroughly triturated with a tissue homogenizer and then placed on ice for 1 h, and the sample was lysed at 14,000 rpm at 4 °C. The mixture was centrifuged for 30 min and the supernatant was collected for further sample preparation. Protein concentrations were detected by BCA Protein Assay Kit (Beyotime, China). The loaded proteins were separated by electrophoresis on 12% sodium dodecyl sulfate-polyacrylamide gels (30 mg total protein/lane). After transfer using PVDF membrane, the membrane was blocked with 5% BSA (Sigma; USA) for 1.5 h at room temperature and then incubated with primary antibody overnight at 4 °C. The next day, the membrane was washed three times for 10 min with TBST, after which the membrane was incubated with horseradish peroxidase-conjugated secondary antibody [goat anti-mouse, goat anti-rabbit (1:10,000, Abcam)] at room temperature. After incubation for 2 h, the membrane was washed three times for 10 min. Then, the membranes were washed three times with 1 × TBST for 10 min each, and the bands were incubated with chemiluminescence reagents and detected by the ChemiDoc™ Touch Imaging System (Bio-Rad, USA). The gray values were analyzed using NIH ImageJ (FIJI) software. The following antibodies were used: rabbit anti-Homer1 (1:1000; #ab184955; Abcam; UK), rabbit anti-RIPK1(1:1000; #DF8234; Affinity; USA), rabbit anti-Phospho-RIPK1(1:1000; #AF2398; Affinity), rabbit anti-RIPK3 (1:1000; #DF10141; Affinity; USA), rabbit anti-Phospho-RIPK3 (1:1000; #AF3894; Affinity; USA), rabbit anti-MLKL (1:1000; #DF7412; Affinity; USA), rabbit anti-Phospho-MLKL (1:1000; #AF3846; Affinity; USA), mouse anti-GAPDH (1:10,000; ab8245; Abcam; UK).

### Cell activity detection

Cell viability was measured using a Cell Count Kit 8 (Beyotime, China). The cell activity was tested and strictly operated according to the manufacturer's instructions. Here, 10 μL detection reagents were added to each well in the 96-well plate, incubated at 37 ℃ for 1 h, and the optical density was measured at an absorption wavelength of 450 nm.

### LDH detection

LDH release was measured according to the manufacturer's instructions (Beyotime). Absorbance was measured at 490 nm using a 96-well microplate reader (Molecular Devices, USA). The LDH release (%) was calculated as the ratio of experimental LDH release to control LDH release.

### ELISA

ELISA was performed in strict accordance with the manufacturer’s instructions. Briefly, we performed gradient dilutions of protein standards, followed by further measurement of the absorbance of the standards at each dilution gradient at an absorption wavelength of 450 nm and the establishment of a standard curve. The cell supernatants were harvested for ELISA. Brain tissue from the ischemic penumbra cortex was used for ELISA. The following ELISA kits were used for detection: Mouse IL-1β ELISA Kit (ab197742; Abcam, UK), Mouse TNFα ELISA Kit (ab108910; Abcam, UK), Mouse TNFR I ELISA Kit (ab202408; Abcam, UK)**,** Mouse Fas Ligand ELISA Kit (ab270894; Abcam, UK).

### Statistical analysis

Statistical analyses were performed using the GraphPad Prism software. Parametric and nonparametric tests were conducted based on the homogeneity of variance. A t test was used to compare the mean variance of homogeneous data, and multiple group comparisons of means were performed using one-way analysis of variance (ANOVA) with the Bonferroni post hoc test. *P* < 0.05 was considered statistically significant.

## Results

### Homer1 expression was upregulated after pMCAO

**Fig. 1:**
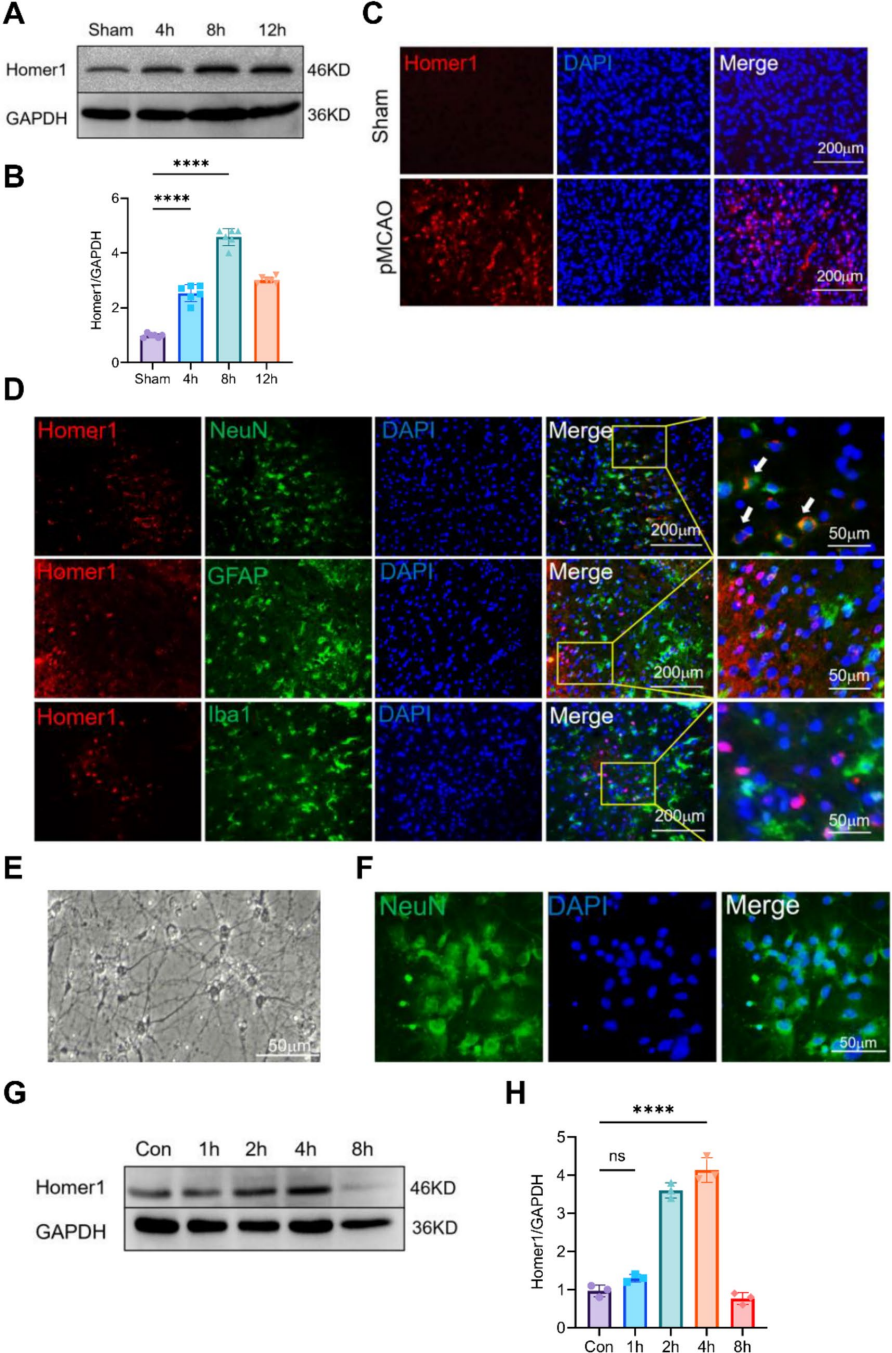
Spatiotemporal expression pattern of Homer1 after pMCAO.** A** Homer1 protein expression of ischemic penumbra cortex after pMCAO 4 h, 8 h, and 12 h. The blots are representative of other replicates in those groups. **B** Quantification of result in **A**. **C** Representative images of Homer1 expression in ischemic penumbra cortex.** D** Immunofluorescence was used to detect the co-localization of Homer1 with neurons, microglia, and astrocytes. **E** Optical microscopic identification images of primary neurons. **F** Immunofluorescence identification of primary neurons. **G** Representative immunoblot of Homer1 in cultured primary neurons after OGD 1 h, 2 h, 4 h, and 8 h. **H** Quantification of result in **G**. For **B**, **H**: ***P < 0.001, and ****P < 0.0001 by one-way-ANOVA analysis. Data are presented as the mean ± SD; *n* = 6/group. All data are representative of three independent experiments

To examine the spatiotemporal expression pattern of Homer1 after pMCAO, pMCAO models were established using C57BL/6 mice. WB results indicated that Homer1 levels in the ischemic penumbra cortex increased from 4 h to a peak at 8 h and then decreased after pMCAO (Fig. 1A, B). We detected the expression of Homer1 at 8 h after pMCAO induction (Fig. 1C). Furthermore, to determine which type of cells in the brain expressed Homer1, we found that Homer1 was mainly located in neurons at the site of the ischemic penumbra cortex tissues through immunofluorescence staining (Fig. 1D).

To verify the expression of Homer1 in vitro, we measured the expression levels of Homer1 in OGD-treated primary neurons. The extracted primary neurons were identified by light microscopy and immunofluorescence (Fig. 1E, F). Compared with the control group (Con), the protein expression level of Homer1 initially increased and then decreased, with peak expression at 4 h after OGD (Fig. 1G, H). Through in vivo and in vitro experiments, Homer1 expression was found to be upregulated after pMCAO, and neurons may be the primary cellular source for Homer1 induction.

### Homer1-KD increased pMCAO-induced necroptosis in vivo and in vitro

**Fig. 2:**
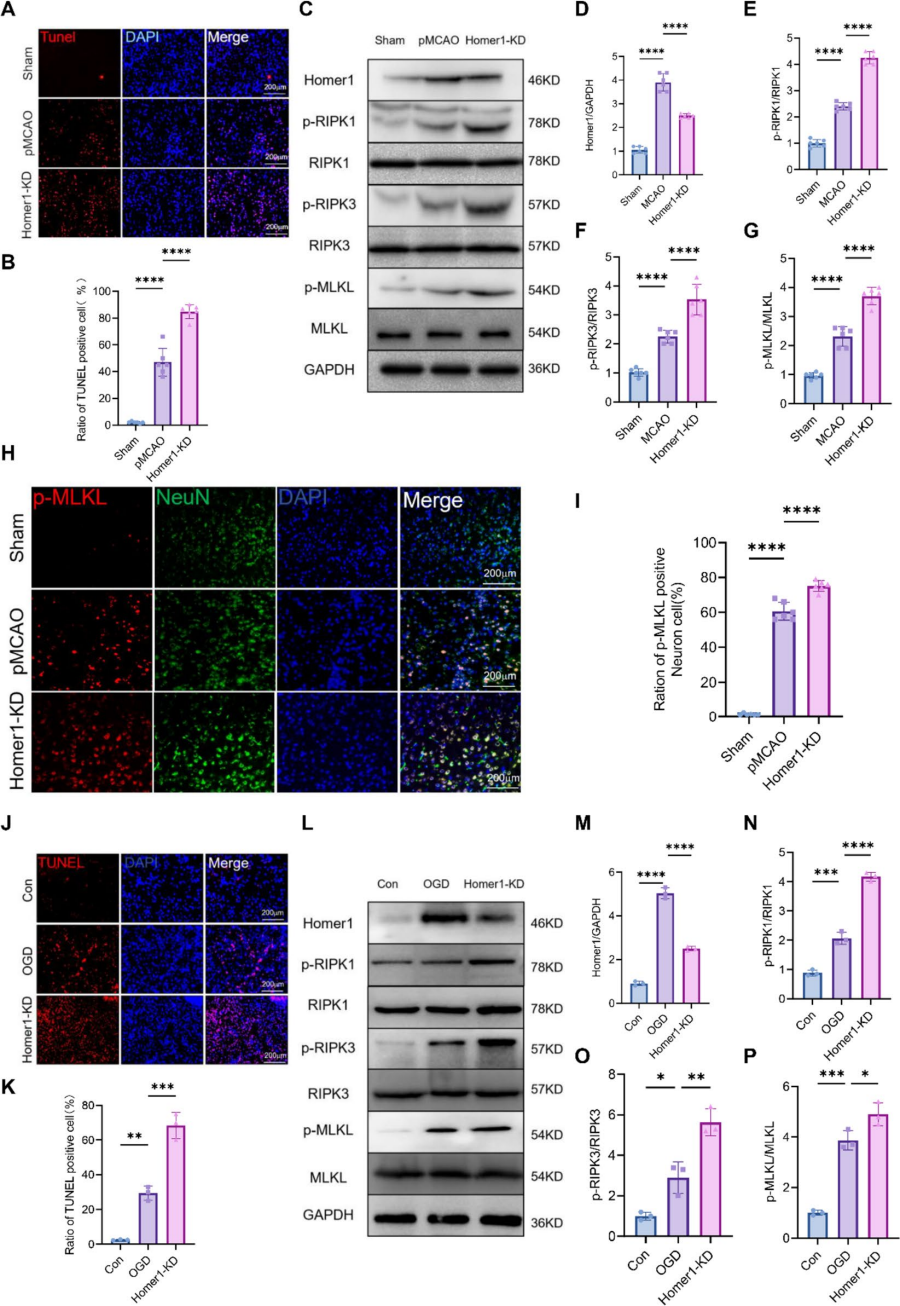
Homer1-KD promoted post-ischemic neuronal necroptosis.** A**, **B** Representative images **A** and quantification **B** of neuronal death based on TUNEL assay in the ischemic penumbra cortex of each group of mice at 8 h after pMCAO. **C** Effects of Homer1-KD on the expression level of p-RIPK1, p-RIPK3, and p-MLKL in the ischemic penumbra cortex of each group at 8 h after pMCAO. **D–G** Quantification of result in **C**. **H** Representative photographs of p-MLKL-positive neurons of brain tissue in each group at 8 h after pMCAO. **I** Quantification of result in **H**. **J**,** K** Representative images **J** and quantification **K** of primary neuronal death based on TUNEL assay in vitro of different groups at 4 h after OGD. **L** Effects of Homer1-KD on the expression level of p-RIPK1, p-RIPK3, and p-MLKL of different groups in vitro experiment at 4 h after OGD. **M–P** Quantification of result in **L**. For **B**, **D**–**G**, **I**, **K**–**N**: *P < 0.05, **P < 0.01, ***P < 0.001, and ****P  < 0.0001 by one-way-ANOVA analysis. Data are presented as the mean ± SD; *n* = 6/group. All data are representative of three independent experiments

Owing to the changes in Homer1 expression induced by pMCAO, we investigated the impact of knockdown Homer1 on the pathological mechanism of necroptosis after pMCAO. First, AAV was used to stably knock down Homer1 in the ischemic penumbra cortex of mice 4 weeks before the pMCAO model. Homer1-KD markedly increased the number of TUNEL-positive cells in the ischemic penumbra cortex tissues for 8 h, demonstrating that Homer1 knockdown accelerated pMCAO-triggered cell death (Fig. 2A, B). Next, we examined key molecules of the necroptosis pathway, and WB (Fig. 2C–G) results showed that Homer1- KD significantly increased p-RIPK1, p-RIPK3, and p-MLKL levels. Additionally, immunofluorescence staining results suggested a significant increase in p-MLKL-positive neurons in the Homer1-KD group compared to the pMCAO group (Fig. 2H, I).

We also examined the effects of Homer1-KD on the necroptosis of primary neurons after OGD in vitro. The effect of Homer1-KD on primary neurons treated with OGD was detected at 4 h by TUNEL staining, and the number of dead cells was significantly higher than that in the OGD group (Fig. 2J, K). WB results suggested that the expression of molecules involved in necroptosis-related pathways was higher in the Homer1-KD group than in the OGD group, which was consistent with the in vivo experiments (Fig. 2L–P). Based on these results, we inferred that Homer1-KD could exacerbate neuronal necroptosis caused by ischemic brain injury.

### Homer1-KD increased pMCAO-induced neuroinflammation in vivo and in vitro

**Fig. 3:**
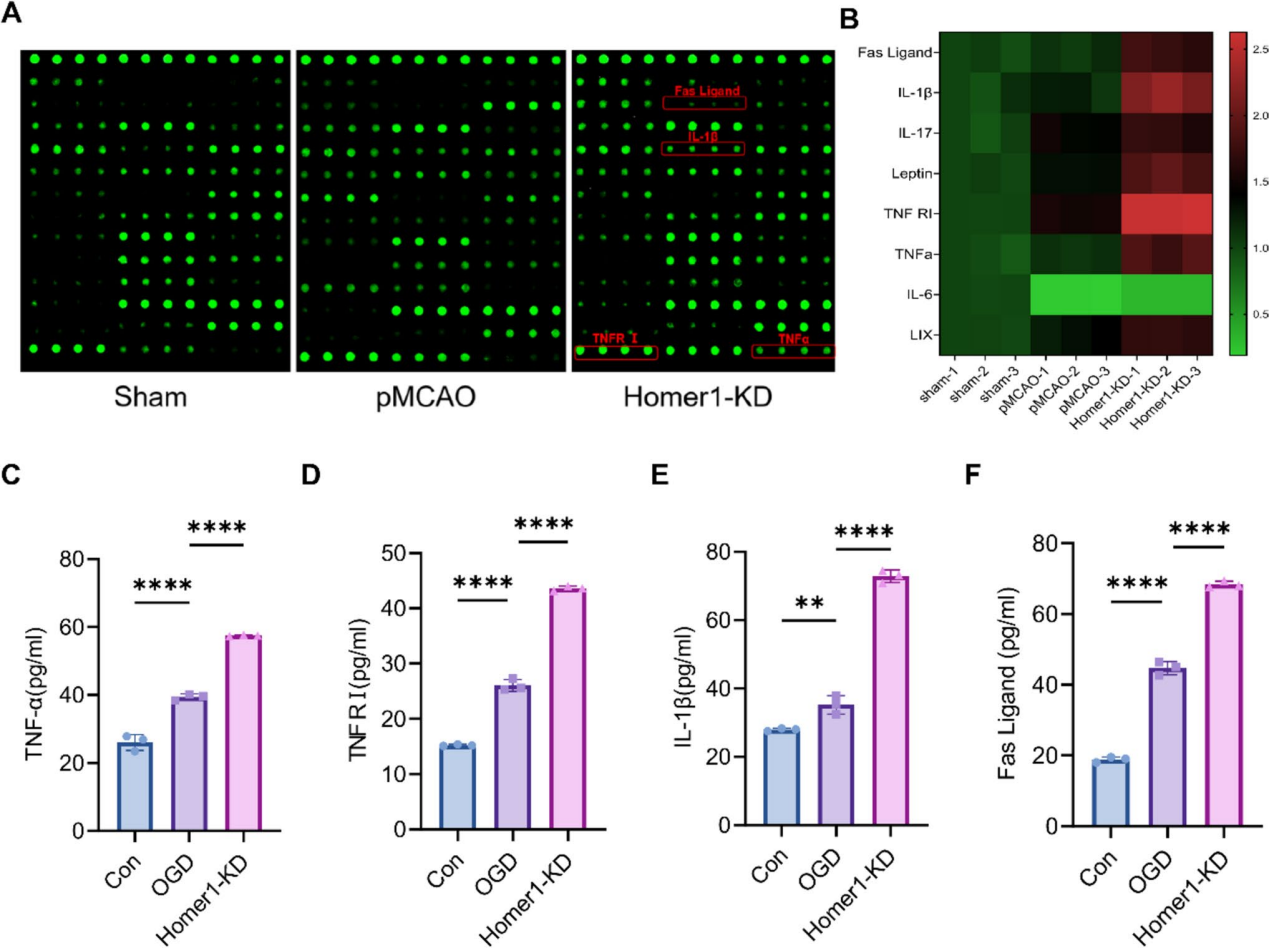
Homer1-KD aggravated proinflammatory cytokine production.** A** Representative experimental diagram of chip slide carrier from ischemic penumbra cortex tissue at 8 h after pMCAO. **B** Heat map of protein chip results.** C** ELISA of TNFα in supernatants of primary neurons in different groups. **D** ELISA of TNFR I in supernatants of primary neurons in different groups. **E** ELISA of IL-1β in supernatants of primary neurons in different groups. **F** ELISA of Fas Ligand in supernatants of primary neurons in different groups. For in vitro cell experiments **C**–**F**, detection time is at 4 h after OGD. For **C**–**F**: **P < 0.01, ****P  < 0.0001 by one-way ANOVA analysis. Data are presented as the mean ± SD; *n *= 3/group. All data are representative of three independent experiments

Given that necroptosis is accompanied by neuroinflammation [25], which plays a crucial role in ischemic stroke [26], we investigated the effects of Homer1-KD on pMCAO-induced neuroinflammation. The ischemic penumbra cortex of the sham, pMCAO, and Homer1-KD groups was used to detect 80 markers on the index protein chips. Further analysis was conducted on the inflammatory factors showing the most significant differences between the pMCAO and Homer1-KD groups (Fig. 3A, B). The results indicated that compared to the pMCAO group, TNFα, TNFR I, IL-1β, Leptin, Fas Ligand, and other proinflammatory factors were significantly elevated in the Homer1-KD group.

Furthermore, inflammatory factors in the supernatants of the control group, OGD, and Homer1-KD cells were tested in vitro. The ELISA detection results indicated that the TNFα, TNFR I, IL-1β, and Fas ligand in the cell supernatant of the Homer1-KD group was higher than that of the pMCAO group (Fig. 3C–F). These data indicated that Homer1-KD exacerbated pMCAO-induced neuroinflammation.

### Homer1-KD accelerated brain injury after pMCAO

**Fig. 4:**
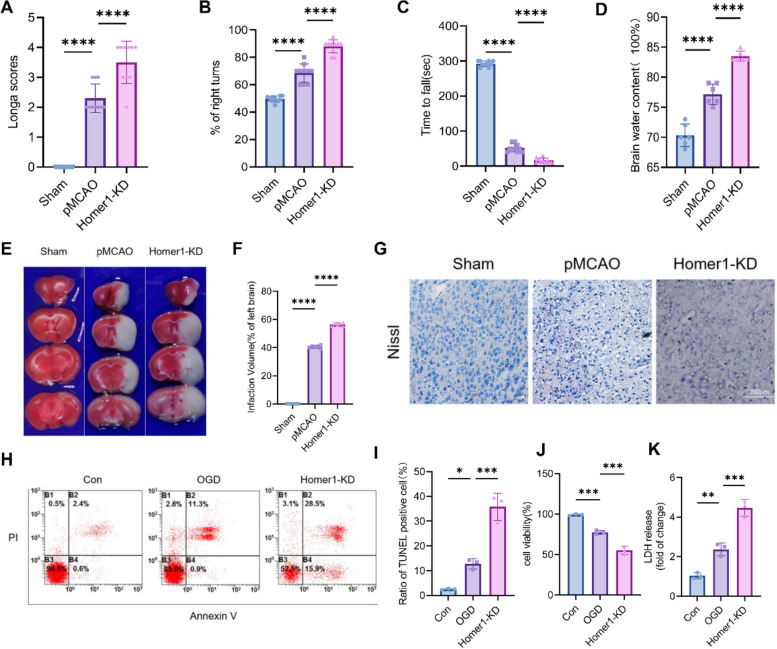
Homer1-KD exacerbated ischemic brain injury. **A** Longa scores of different groups. **B** Corner test of different groups.** C** Rota-rod test of different groups. **D** Detection of brain water content. **E** TTC-stained sections of different groups of mice. **F** Quantification of the infarct volume. **G** Representative photographs of Nissl staining of brain tissue in each group. **H** Apoptosis rate of different modified primary neuron cells by flow cytometry. **I** Quantification of result in **H**. **J** Cell viability test in different groups. **K** LDH release detection in different groups. Cell viability **J** or LDH release **K** was assessed and quantified for transfected neurons or without treatment (Con). The primary cultured neurons were transfected with plasmid for 2 days before cell viability or LDH release assay. For in vivo experiment **A**–**G**, detection time is at 8 h after pMCAO. For in vitro experiment **H**–**K**, detection time is at 4 h after OGD. For **A**–**D**, **F**, **I**, **J**, **K**: **P < 0.01, ***P < 0.001, and ****P < 0.0001 by one-way ANOVA analysis. Data are presented as the mean ± SD; *n* = 6/each group and neurological scores *n* = 10/each group. All data are representative of three independent experiments

Considering the destructive role of Homer1 knockdown in necroptosis, we investigated whether Homer1 knockdown exerts a direct effect on ischemic brain injury and neurological deficits. Compared to the pMCAO model group, significant aggravation of neurological deficits was observed at 8 h in the Homer1-KD group (Fig. 4A–C), and brain water content was significantly increased (Fig. 4D) in the Homer1-KD group. TTC staining indicated that the infarct area was markedly enhanced in the Homer1-KD group at 8 h compared to the pMCAO group (Fig. 4E, F). Nissl staining showed that Homer1-KD aggravated the activity of neurons at the ischemic site compared to the pMCAO group (Fig. 4G).

Furthermore, we conducted OGD treatment of primary neurons in vitro to simulate the microenvironment of ischemic brain injury. Flow cytometry analysis showed that, compared to the OGD group, primary neurons transfected with the Homer1-KD plasmid showed more cell death (Fig. 4H, I). Homer1-KD reduced cell viability (Fig. 4J) and increased LDH release (Fig. 4K) 4 h after OGD. Together, these results suggested that the knockdown of Homer1 was followed by the aggravation of pMCAO-induced damage in vivo, as well as OGD-induced damage in vitro.

### Homer1 protein inhibited necroptosis-induced brain injury and neuroinflammation after pMCAO in Homer1^flox/flox^/Nestin-Cre^+/−^ mice

**Fig. 5:**
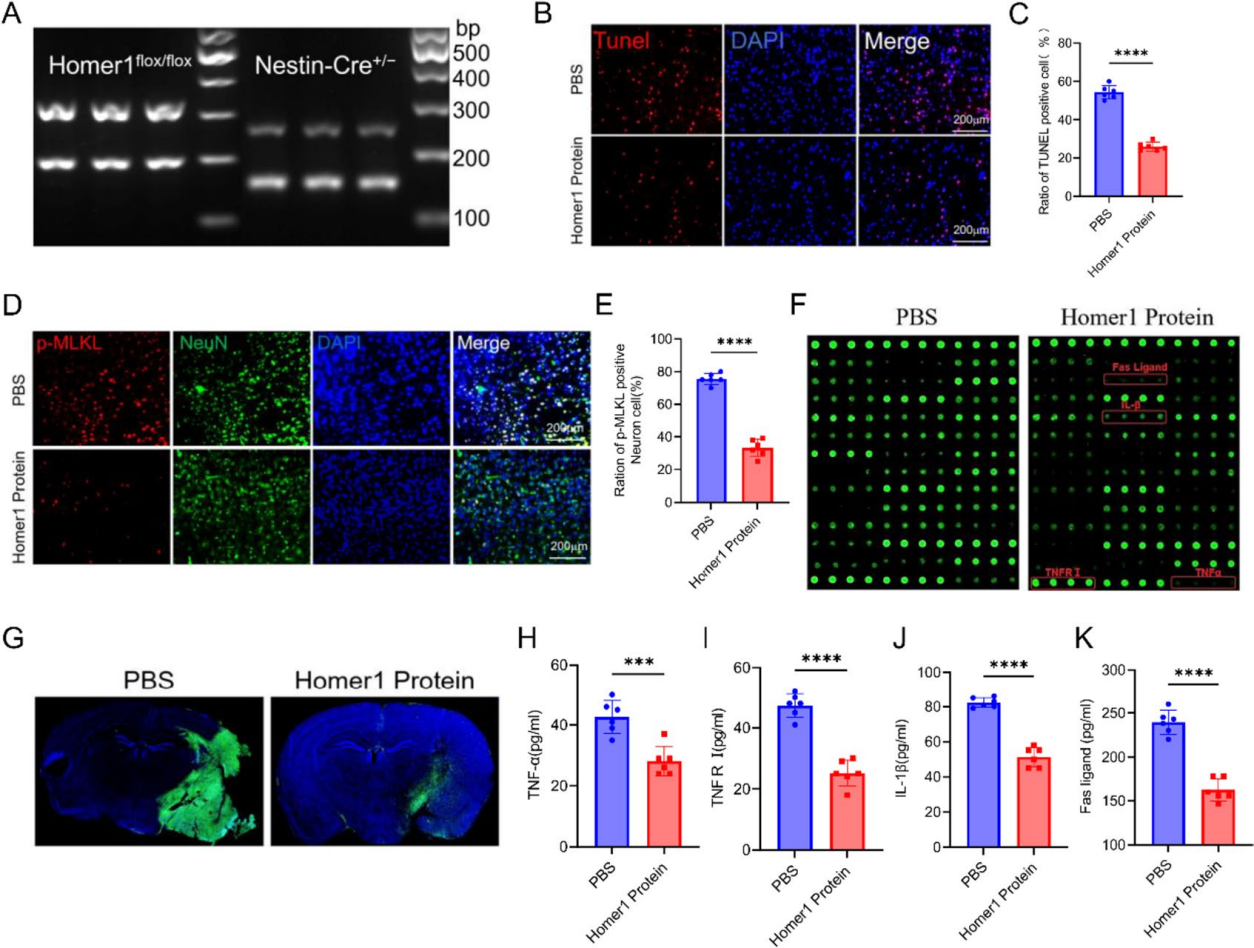
Homer1 protein ameliorated the pathological indexes of pMCAO.** A** Genotype identification of Homer1^flox/flox^/ Nestin-Cre^+/−^ mice. **B** Representative photographs of Tunel staining of brain tissue in each group. **C** Quantification of result in **B**. **D** Representative photographs of p-MLKL-positive neurons of brain tissue in each group. **E** Quantification of result in **D**. **F** Representative experimental diagram of chip slide carrier. **G** IgG staining for blood–brain barrier damage. **H** ELISA of TNFα of ischemic penumbra cortex in different groups. **I** ELISA of TNFR I of ischemic penumbra cortex in different groups. **J** ELISA of IL-1β of ischemic penumbra cortex in different groups. **K** ELISA of Fas ligand of ischemic penumbra cortex in different groups. All brain tissues in the experiment were taken from ischemic penumbra cortex tissue at 8 h after pMCAO. For **C**, **E**, **H**–**K**: *P < 0.05, **P < 0.01, and ***P < 0.001 by Student’s *t* test. Data are presented as the mean ± SD; *n* = 6/group. All data are representative of three independent experiments

Previous studies showed that Homer1 knockdown exacerbated neuronal necroptosis and neuroinflammation during ischemic brain injury. Therefore, *Homer1* conditional knockout mice (Homer1^flox/flox^/Nestin-Cre^+/−^) were constructed to investigate the protective role of Homer1 protein in ischemic brain injury. First, regarding the impact of the conditional knockout of the *Homer1* gene on neural function in mice without the pMCAO model, we conducted behavioral tests on wild-type and conditional knockout Homer1 mice. The results showed that there were no statistically significant differences in the behavioral scores (Longa score, rota-rod test, and corner test) of mice in each group, indicating that knocking out Homer1 did not have an impact on neurological deficits in the mice (Supplementary Fig. 1). In two sets of Homer1^flox/flox^/Nestin-Cre^+/−^ mice after pMCAO, one group was injected with PBS (20 μL) and the other group was injected with Homer1 protein (1 μg, dissolved in 20 μL PBS) (1.43 mm posterior and 3 mm lateral to the bregma) (depth, 2.0 mm from the bone surface) 5 min after pMCAO. Genotypes of Homer1^flox/flox^/Nestin-Cre^+/−^ mice with conditional gene knockout were identified by PCR (Fig. 5A). TUNEL staining showed that injection of Homer1 protein significantly ameliorated cell death (Fig. 5B, C). Moreover, compared to the PBS group, the Homer1 protein group showed a significant decrease in the number of p-MLKL-positive neurons (Fig. 5D, E).

We tested the inflammatory spectrum of the ischemic penumbra cortex and found that after in situ injection of Homer1 protein, the expression of inflammatory factors was significantly reduced (Fig. 5F). IgG staining results suggested that compared to the PBS group, the Homer1 Protein group had significantly reduced damage to the blood–brain barrier after pMCAO (Fig. 5G). The main inflammatory factors in the ischemic penumbra cortex of the mice were detected using ELISA. ELISA results suggested that the Homer1 Protein group showed significant reduction in TNFα, TNFR I, IL-1β, and Fas ligand compared to the PBS group (Fig. 5H–K).

### Homer1 protein ameliorated neurological deficits and outcomes in Homer1^flox/flox^/Nestin-Cre^+/−^ mice after pMCAO

**Fig. 6:**
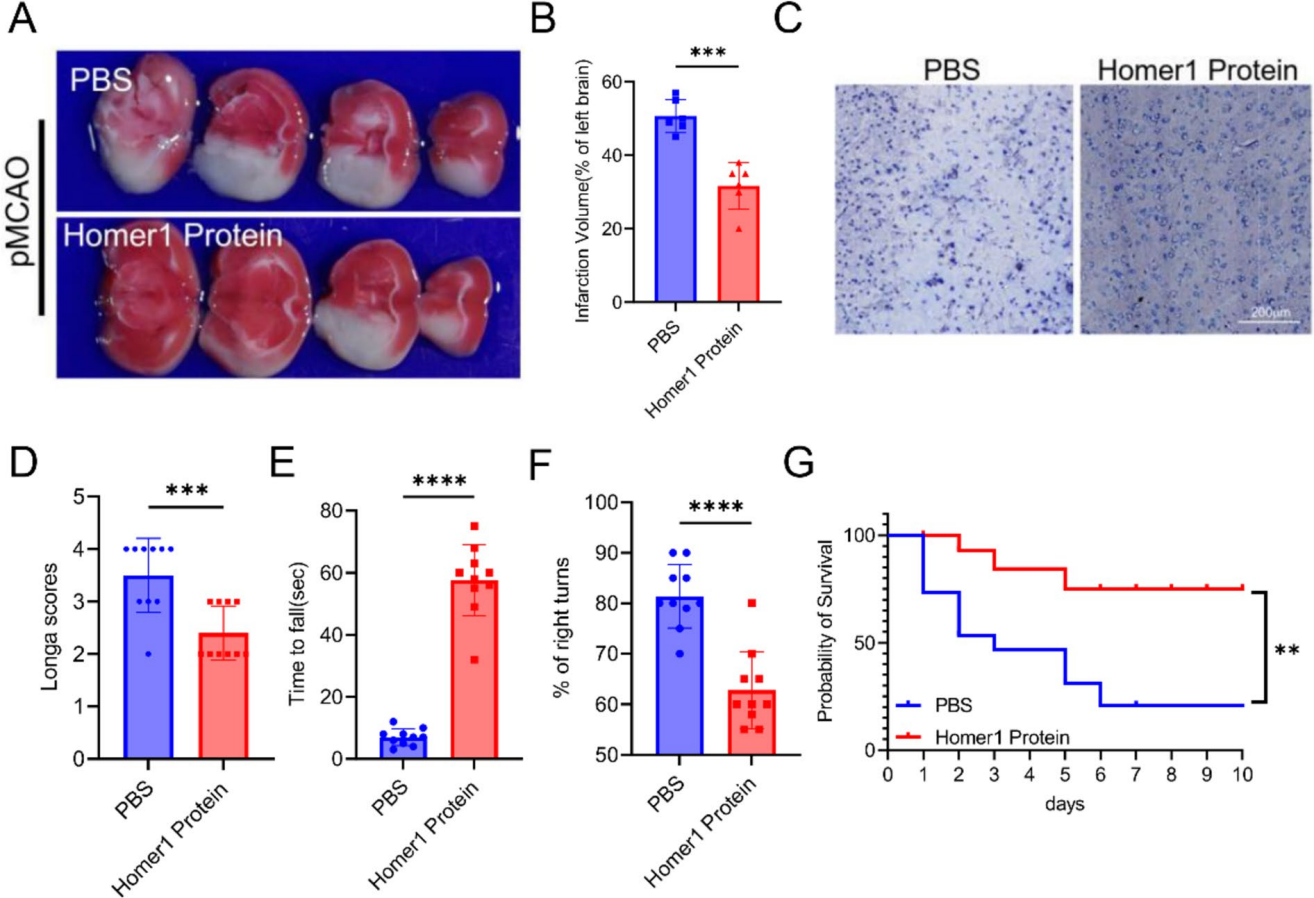
Homer1 protein ameliorated neurological deficits and outcomes of pMCAO.** A** Representative photograph of TTC-stained sections of different groups of mice at 8 h after pMCAO. **B** Quantification of result in **A**. **C** Representative photographs of Nissl staining of ischemic penumbra cortex in each group. **D** Longa scores of different groups. **E** Rota-rod test of different groups. **F** Corner test of different groups. **G** Survival curve of mice in each group (*n* = 15) after pMCAO. For in vivo experiment (**A**–**F**), testing time is at 8 h after pMCAO. For **B**, **D**–**G**: **P < 0.01, and ***P < 0.001 by one-way-ANOVA analysis. Data are presented as the mean ± SD; TTC stain and Nissl stain *n* = 6/each group, neurological scores *n* = 10/each group, and survival time *n* = 15/each group. All data are representative of three independent experiments

Previous experimental results confirmed that Homer1 protein can alleviate the pathological process of necroptosis in Homer1^flox/flox^/Nestin-Cre^+/−^ mice. Additionally, we examined the infarct volume and neurological impairment in mice as well as the effect of Homer1 protein on the survival time of mice after pMCAO. TTC staining showed a significant reduction in infarct size in the Homer1 protein group compared to that in the PBS group (Fig. 6A, B). Moreover, the Nissl staining results indicated a significant recovery of neuronal activity in the ischemic area of the Homer1 protein group (Fig. 6C). We also conducted behavioral experiments to detect changes in neurological deficits in mice. The results of the Longa, corner, and rota-rod tests suggested that the neurological deficit symptoms in the Homer1 Protein group were significantly ameliorated compared to those in the PBS group (Fig. 6D–F). At the same time, we also used 30 Homer1^flox/flox^/Nestin-Cre^+/−^ mice (15 mice in each group) and observed the survival time of the mice in both groups. The results showed that the survival time of mice in the Homer1 Protein group was significantly prolonged compared to that of mice in the PBS group (Fig. 6G).

## Discussion

In the early stages of ischemic stroke, the expression of Homer1 in the ischemic penumbra cortex was highest at 8 h and was mainly expressed in neurons. Homer1 knockdown resulted in the aggravation of neuronal necroptosis and inflammatory reactions, enlargement of the cerebral infarction area, and further aggravation of neurological deficit symptoms in mice. Furthermore, in the *Homer1* conditional knockout mouse pMCAO model, application of Homer1 protein reduced neuronal necroptosis and proinflammatory factor secretion, promoting functional recovery in mice. Therefore, we conclude that Homer1 can ameliorate the prognosis of ischemic stroke by reducing necroptosis-induced brain injury and neuroinflammation.

In the present study, it was found that Homer1 expression was upregulated and peaked at 8 h after pMCAO in vivo. The expression of Homer1 first increased and then decreased, reaching its peak at 4 h in vitro,. Therefore, 8 h after pMCAO and 4 h after OGD were used as the follow-up experimental treatments. In both in vitro and in vivo experiments, Homer1 expression first increased, peaked, and then decreased. A possible reason was that when the body experiences ischemia, the self-defense mechanism was triggered, and Homer1, as a protective molecule, may increase to a certain extent, slowing the degree of damage. However, when ischemic injury persisted, the compensatory mechanisms of the body was exhausted, causing the expression of Homer1 to gradually decrease.

During the MCAO model construction process, we optimized the pMCAO model by inserting a silicone-coated nylon suture into the right middle cerebral artery, according to the bifurcation of the right CCA and the anatomical position of the ICA. After optimization, our ischemic model still had the following advantages without affecting ischemia in mice. First, the procedure of ligating the right external carotid artery was abandoned, which could avoid the fluctuation of blood pressure and heart rate of mice caused by repeated squeezing and touching of the carotid sinus during the experimental process and greatly reduced the mortality of mice. Second, the optimized operating method reduces the surgical wound size and minimizes mechanical damage to blood vessels and peripheral nerves, avoiding unnecessary damage as much as possible. In addition, during the modeling process, we found that a small number of mice exhibited neurological deficits accompanied by "whitening" of the ipsilateral eye, similar to the symptoms of decreased vision in the ipsilateral eye of some patients with anterior circulation cerebral infarction in clinical practice [27]. This suggests that, while paying attention to cerebral infarction, we should also pay attention to ipsilateral eye perfusion in patients. This may also provide a new application for the model.

Resident immune cells in the central nervous system and peripheral immune cells that infiltrate the broken blood–brain barrier can secrete many inflammatory factors [28, 29]. In this study, we used inflammation chips to examine the expression of inflammatory factors in ischemic brain tissues. It was found that TNFα and TNFR I expression significantly increased after the knockdown of Homer1. In the rescue experiment, Homer1 protein can inhibit the expression of proinflammatory factors and ameliorate the degree of brain damage in mice. This confirms the role of TNFα and its receptor-mediated necroptosis [30] and suggests that Homer1 may play a protective role in it. At the same time, we also found that Fas Ligand expression was upregulated after Homer1 knockdown. Fas ligand is a member of the TNF protein family and can cause necroptosis after activation [31]. Elevated Fas and FasL levels have been reported in damaged brain tissue in various neurological disorders, including cerebral ischemia [32]. Notably, we also found that, compared to the ordinary pMCAO model group, the Homer1 knockdown group showed a significant increase in leptin, suggesting that leptin may play a role as an anti-inflammatory factor. Leptin contributes to atherosclerosis by triggering inflammatory responses and oxidative stress and enhancing platelet aggregation and migration [33]. However, intracerebral injection of leptin can ameliorate the permeability of the blood–brain barrier, inhibit the activation of matrix metallopeptidase 9 (MMP-9) [34]. Different studies have shown different results in terms of protection or damage, which requires further research. In this study, we found that inflammatory factors such as tumor necrosis factor, IL-1β, and leptin increased with the knockdown of Homer1, and TTC results indicated a further increase in infarct size. Injecting Homer1 protein into the brain of Homer1^flox/flox^/Nestin Cre^+/−^ mice can significantly inhibit the expression of these proinflammatory factors. However, it is currently unknown whether Homer1 can directly regulate the activation of central or peripheral inflammatory cells, which is the direction for further research in the next stage of this project.

Currently, clinical treatments for acute ischemic brain injury mainly include intravenous thrombolysis, thrombectomy, and drug therapy [35]. Common drugs used include antiplatelet drugs and lipid-lowering agents [36]. However, no drugs are available for treating dying cells or neuroinflammation in the ischemic region. In this study, stereotaxic injection of Homer1 into the middle cerebral artery blood supply area of mice showed that Homer1 significantly diminished cell death and neuroinflammation, reduced the cerebral infarction area, and ameliorated the symptoms of neurological deficits in mice. In situ administration avoids the problem of drugs not crossing the blood–brain barrier or being broken down due to liver metabolism. However, if the Homer1 protein is administered intravascularly, similar to most drugs in clinical practice, whether there are pharmacodynamic changes after hepatic metabolism and toxic effects remain to be further investigated. Additionally, as there is currently no murine Homer1 protein on the market, we used the human Homer1 protein; therefore, species variation is an issue that must be considered.

Our results indicated that Homer1 is significantly expressed in the ischemic penumbra cortex of the brain. Knockdown of Homer1 accelerated necroptosis-induced brain injury and neuroinflammation. Homer1 ameliorates brain injury induced by ischemic stroke in conditional knockout mice. Therefore, Homer1 may be a novel and promising therapeutic target for the development of effective treatments for ischemic brain injury. However, the current study remains limited, and further studies are needed to reveal the translational potential of Homer1 in patients with ischemic stroke.

### Supplementary Information

Below is the link to the electronic supplementary material.

Supplementary file1 (DOCX 107 KB)

## Data Availability

The data sets and materials produced during the present study are available from the corresponding authors upon reasonable request.

## Abbreviations

Homer1: Homer scaffold protein 1
pMCAO: Permanent middle cerebral artery occlusion
CCA: Common carotid artery
ECA: External carotid artery
ICA: Internal carotid artery
OGD: Oxygen and glucose deprivation
AAV: Adeno-associated virus
KD: Knockdown
TTC: Triphenyl tetrazolium chloride
WB: Western blot
RIPK1: Receptor-interacting protein kinase 1
RIPK3: Receptor-interacting protein kinase 3
MLKL: Mixed lineage kinase domain-like protein
TNF-α: Tumor necrosis factor-α
IL-1β: Interleukin-1β
