# Supplementary material for: Homer1 ameliorates ischemic stroke by inhibiting necroptosis-induced neuronal damage and neuroinflammation
Source: Inflamm Res. 2023 Dec 13;73(1):131–44. doi: 10.1007/s00011-023-01824-x (PMC10776472; doi:10.1007/s00011-023-01824-x)
Supplement: Supplementary file 1 — Supplementary file1 (DOCX 107 KB) [file 11_2023_1824_MOESM1_ESM.docx]

**Supplementary Figure**


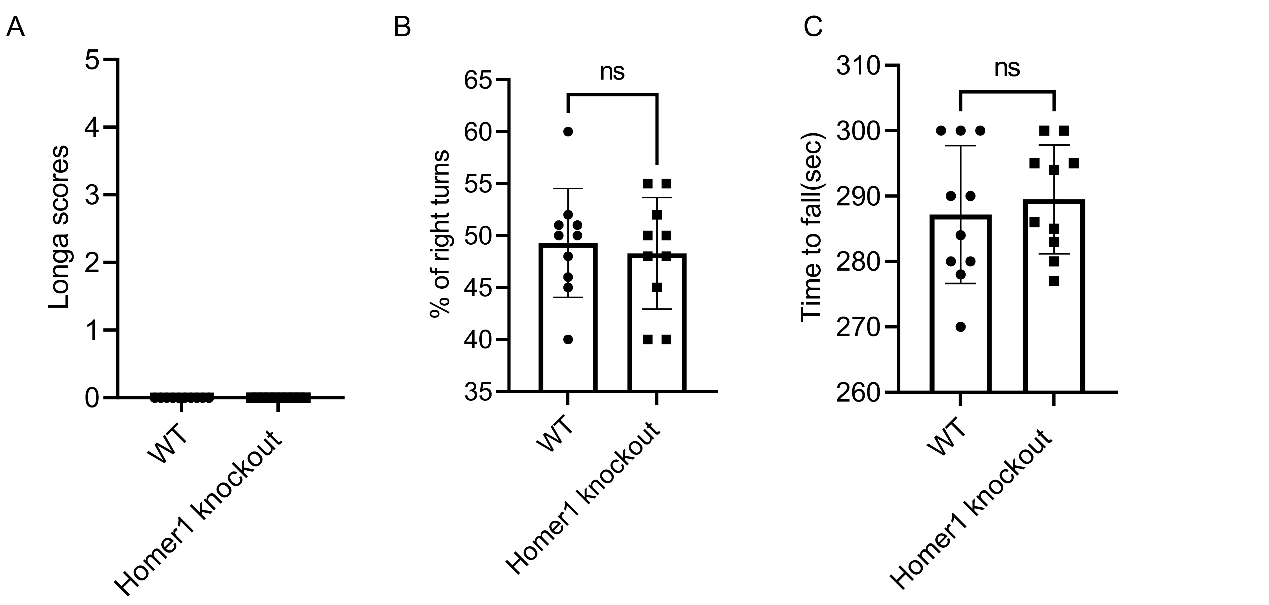


Supplementary Fig. 1. **A** Longa scores of wild-type mice (WT) and Homer1 conditional knockout mice. **B** Corner Test of wild-type mice (WT) and Homer1 conditional knockout mice. **C** Rota-Rod Test of wild-type mice (WT) and Homer1 conditional knockout mice. Data are presented as the mean ± SD; neurological scores n=10/each group. All data are representative of three independent experiments.
